# Effects of the Targeted Regulation of CCRK by miR-335-5p on the Proliferation and Tumorigenicity of Human Renal Carcinoma Cells

**DOI:** 10.1155/2022/2960050

**Published:** 2022-10-14

**Authors:** Xiaojia Zuo, Chaojun Lu, Yanjun Zheng, Donglin Lai, Dingsheng Liu, Guoqing Wan, Changlian Lu, Xuefeng Gu

**Affiliations:** ^1^Shanghai Key Laboratory of Molecular Imaging, Zhoupu Hospital, Shanghai University of Medicine and Health Science, Shanghai, China; ^2^Operation Room, Huashan Hospital Affiliated to Fudan University, Shanghai, China; ^3^Shanghai Gongli Hospital, The Second Military Medical University, Shanghai, China; ^4^School of Medical Medicine, Guizhou Medical University, Guiyang, Guizhou, China; ^5^School of Medical Instruments and Food Engineering, University of Shanghai for Science and Technology, Shanghai, China; ^6^School of Pharmacy, Shanghai University of Medicine and Health Sciences, Shanghai, China

## Abstract

Cell cycle-related kinase (CCRK) is most closely related to cyclin-dependent protein kinase, which may activate cyclin-dependent kinase 2 and is associated with the growth of human cancer cells. However, the expression and function of CCRK in the pathogenesis of clear cell renal cell cancer (ccRCC) are unclear. Herein, this research aimed to explore the potential mechanism of the targeted regulation of CCRK by miR-335-5p on the proliferation and tumorigenicity of human ccRCC cells. The results showed that CCRK was significantly overexpressed in ccRCC tissues and cells, and knockdown of the CCRK expression by shRNA inhibited cell proliferation *in vitro* and *in vivo* and enhanced cell apoptosis *in vitro*, which indicated that CCRK could be a potential target for antitumour drugs in the treatment of ccRCC. Moreover, miR-335-5p was found to bind directly to the 3′ untranslated region of CCRK, was expressed at markedly low levels in ccRCC cells, and was closely associated with the tumour stage. The overexpression of CCRK partially reversed the inhibitory effects of miR-335-5p on the cell growth of ccRCC, which implied that miR-335-5p could serve as a promising tumour inhibitor for ccRCC. In summary, CCRK could serve as an alternative antitumour drug target, and miR-335-5p could be a promising therapeutic tumour inhibitor for ccRCC treatment.

## 1. Introduction

Renal carcinoma is one of the most common malignant tumours of the urinary tract, with over 400,000 new cases diagnosed and over 170,000 renal carcinoma-related deaths worldwide each year [1–3]. Renal cell carcinoma (RCC), the most prevalent form of renal carcinoma, originates from renal tubular epithelial cells and occupies over 90% of renal carcinoma cases [3, 4]. RCC encompasses more than 10 histological and molecular subtypes, of which clear cell RCC (ccRCC) is one of the most common subtypes, accounting for 65–70% of RCC cases [3]; ccRCC is characterized by high mortality, invasion, and metastasis [3]. Considering the poor survival rate and prognosis of ccRCC, it is essential to be diagnosed and treated in the early stage of patients with ccRCC. Thus, it is necessary to reveal the underlying molecular mechanisms involved in the pathogenesis and progression of ccRCC and to seek new therapies to improve the prognosis of patients with advanced-stage disease.

Cell cycle-related kinase (CCRK), also known as cyclin-dependent kinase 20 (CDK20) or p42, a member of the CDK family, was first identified in HeLa cells in 2000 [5, 6]. Increasing studies have indicated that CCRK is closely associated with human cancers [7, 8]. CCRK is ubiquitously expressed in cells originating from various tumour tissues, but its expression is also significantly upregulated in lung, brain, colorectum, liver, and ovary cancers [9–12]. Such aberrant expression of CCRK is usually positively correlated with histopathological grade, advanced tumour stage, shorter patient survival, and poor prognosis, suggesting a vital role of CCRK in the pathogenesis and prognosis of human tumours [13]. CCRK is involved in various kinds of cell signalling pathways associated with the genesis and development of cancer, such as cell cycle and apoptosis pathways [14]. These findings suggest that CCRK is a promising target in the antitumour therapy. However, the expression and function of CCRK in the pathogenesis of ccRCC remain unknown.

MicroRNAs (miRNAs) are highly conserved, small noncoding RNA molecules that are 17–25 nt in length, and they were first described in 1993 [15, 16]. miRNAs play a pivotal role in regulating gene expression at the posttranscriptional level by selectively and specifically binding to a target mRNA, resulting in mRNA translational inhibition or degradation [3, 17]. It has been shown that miRNAs regulate multiple cellular processes, including cell differentiation, proliferation, apoptosis, metastasis, and cell cycle progression [3, 18–20]. Many miRNAs, such as miR-191, miR-139-5p, and miR-29a, are involved in the development of cancer and have been shown to act as biomarkers, oncogenes, or tumour inhibitors [16, 21, 22]. More importantly, the target genes of these miRNAs and their underlying mechanisms in various human cancers have been revealed [21, 22].

It has been reported that miR-335-5p is expressed at low levels in various human tumours, including colorectal cancer, pancreatic cancer, uterine leiomyoma, gallbladder cancer, breast cancer, gastric cancer, and epithelial ovarian cancer [23–29], and it may play a role as a tumour inhibitor. Recent studies reported miR-335-5p is associated with RCC [3, 30]. However, the role of miR-335-5p and CCRK in the pathogenesis of ccRCC has not yet been determined. Here, this study focused on exploring the potential mechanism of the targeted regulation of CCRK by miR-335-5p on the proliferation and tumorigenicity of human ccRCC cells. The results revealed that CCRK could serve as an alternative antitumour drug target, and miR-335-5p could be a promising therapeutic tumour inhibitor for ccRCC treatment.

## 2. Materials and Methods

### 2.1. Data Obtained

Clinical data, including 110 ccRCC tissues and 84 normal tissues, were downloaded from the Clinical Proteomic Tumour Analysis Consortium (CPTAC, https://proteomics.cancer.gov/programs/cptac). miRNA data, including 239 ccRCC tissues and 69 normal tissues, were analysed online with UALCAN (https://ualcan.path.uab.edu/index.html). This work was approved by the Ethics Committee of Shanghai University of Medicine and Health Sciences Affiliated Zhoupu hospital [31].

### 2.2. Cell Culture and Transfection

Human ccRCC cell lines (A498, 786-O, Caki-1, and ACHN cells) were purchased from ATCC (https://www.atcc.org/). The A498, Caki-1, and ACHN cell lines were cultured in EMEM. The 786-O cell line was cultured in RPMI-1640 medium. All the media were supplemented with 10% fetal bovine serum (Gibco, Waltham, MA) and 1 : 1 penicillin/streptomycin (final concentration of 100 U/mL) and incubated at 37°C in 5% CO_2_.

CCRK (NM_178432.1) short hairpin RNA (shRNA), control scrambled shRNA (scr shRNA), and overexpression plasmids were designed and synthesized by RioScience (Shanghai, China). The target sequence of CCRK was 5′-GAAGGTGGCCCTAAGGCGGTTGGAAGACG-3′. Human miR-335-5p mimic, miR-335-5p inhibitor, and the corresponding controls were synthesized by GenePharma (Shanghai, China). To explore the function of CCRK, a rescue experiment was performed in A498 cells with CCRK knockdown. The CCRK plasmid (pcDNA3.1) was transfected into A498 cells after CCRK knockdown using Lipofectamine 2000 reagent (Invitrogen, CA, USA) in six-well plates.

### 2.3. Immunohistochemistry

The immunohistochemistry with the antibody HPA027379 against human CCRK tissue sections were obtained from the protein atlas (https://www.proteinatlas.org/ENSG00000156345-CDK20/pathology/renal+cancer). The expression of Ki-67 protein in mice tumour tissue was detected by immunohistochemistry to evaluate cell proliferation of the transplanted tumour *in vivo*. The transplanted tumour tissues of mice were routinely embedded in paraffin, and the sections were stained according to the protocols of immunohistochemical detection kit. The sections were added a drop of Ki-67 primary antibody (1 : 500) and incubated overnight at 4°C, then biotinylated secondary antibody was incubated at 37°C for 30 minutes. Then, the sections were incubated with streptavidin peroxidase from Streptomyces avidinii (Sigma-Aldrich#S5512) at 37°C for 30 minutes and colour rendered using the DAB chromogenic reagent, counterstained by hematoxylin, differentiated by hydrochloric acid ethanol, dehydrated, made transparent and sealed, and observed under optical microscope. The percentage of positive cells represented the proliferation index.

### 2.4. Real-Time Quantitative PCR

Total RNA was extracted from the cells with TRIzol™ (Cat#15596018, Invitrogen, USA). Reverse transcription PCR was performed using a one-step RNA PCR kit (Cat#RR064B, TaKaRa, China). SYBR Green Supermix kit (C11733046, Invitrogen, USA) was used to perform real-time quantitative PCR (RT-qPCR) on an ABI PRISM® 7500 sequence detection system. The primer sequences used for RT-qPCR were synthetized by Saiyin Biotechnology (Shanghai) Co., Ltd., and glyceraldehyde-3-phosphate dehydrogenase (GAPDH) was used as the internal reference. The sequences of the primers used for RT-qPCR were as follows: CCRK-Forward: 5′-CCTCCATCAGTACTTCTTCACA-3′; CCRK-Reverse: 5′-GAATCAGCTCTGGGTTCAAC-3′; miR-335-5p Forward: 5′-ACACTCCAGCTGGGTCAAGAGCAATAACGAAA-3′; miR-335-5p Reverse: 5′-CTCAACTGGTGTCGTGGA-3′; and miR-335-5p RT primer: 5′-CTCAACTGGTGTCGTGGAGTCGGCAATTCAGTTGAGACATTTTTC-3′. Every experiment was repeated thrice.

### 2.5. Western Blotting

Total protein was extracted from cells with radioimmunoprecipitation assay (RIPA) buffer (Thermo Fisher Scientific, Waltham, MA), and the concentration of the total protein was measured with a bicinchoninic acid (BCA) kit (Yeasen, Shanghai, China). Next, equal amounts of proteins were separated by 10% SDS-PAGE and transferred onto the PVDF membranes. Subsequently, the PVDF membranes were blocked with 5% nonfat milk for 2 h and incubated overnight at 4°C with the following diluted primary antibodies: anti-caspase-3 (Cat#9665S), anti-cleaved caspase-3 (Cat#9579S), anti-cyclin D1 (Cat#2922S), anti-Bax (Cat#2772S), and anti-*β*-actin (Cat#4967). Then, the PVDF membranes were further incubated with HRP-labelled goat anti-rabbit immunoglobulin G antibodies (Abcam, ab6721) for 2 h. Next, ECL luminescent (Cat#36208ES60, Yeasen, Shanghai, China) was used to visualize the colour of the PVDF membranes. Images of the PVDF membranes were obtained by a Bio-Rad image analysis system (Bio-Rad, Richmond, CA, USA), and the quantification of the target proteins was performed with ImageJ software [32].

### 2.6. CCK-8 Assay and Colony Formation Assay

Cell proliferation was assessed using a CCK-8 assay. In brief, 2 × 10^3^ cells were seeded in 96-well plates. Then, after 1, 2, 3, 4, and 5 days, the media were replaced with fresh media containing 10% CCK-8 solution, and the cells were incubated for 2 h. The cell concentrations in the 96-well plates were evaluated based on the absorbance measured at 450 nm. For the colony-forming assay, cells were seeded in 6-well plates at 2 × 10^2^ cells/well and incubated for 2 weeks. Then, the colonies were washed with PBS, fixed with absolute ethyl alcohol, and stained with 0.5% crystal violet. The colonies that turned blue were considered positive, and the cells of blue colonies were counted under an inverted microscope (Olympus, Japan).

### 2.7. Flow Cytometry Assay

Cells were cultured, collected, fixed at 4°C overnight, and then washed with PBS. A total of 0.1 ml cell suspension (1.0 × 10^6^ cell/ml) was stained with propidium iodide (Cat#P34813, ABCONE, Shanghai, China) in the dark for 30 min at 4°C, and the stained cells were filtered through a 50 *μ*m nylon mesh and routinely washed. Cell cycle progression and apoptosis were assessed by flow cytometry (FACSCalibur, Becton Dickinson), and the data obtained by flow cytometry were further analysed by FlowJo software (Tree Star, USA) to calculate the cell proliferation index.

### 2.8. Tumorigenesis Assay

The animal protocols [33] were approved by the Animal Experiments Ethics Committee of Shanghai University of Medicine and Health Sciences Affiliated Zhoupu Hospital. In brief, A498 cells (transfected with CCRK-shRNA or scr-shRNA) were transplanted into the subcutaneous tissue of 6-week-old male BALB/c nude mice (Shanghai Sippr BK Laboratory Animals Ltd., Shanghai, China) at a concentration of 2 × 10^6^ cells/mL (*n* = 4 mice per group). Then, the tumour growth in mice was monitored every 2 days, and the tumour volume was calculated with the following formula: Tumour Volume = (length × width^2^) × 0.5. The mice were sacrificed, and the tumours were harvested on day 27.

### 2.9. Dual-Luciferase Reporter Assay

Fragments of the 3′-untranslated region (UTR) of CCRK containing putative binding sequences of miR-335-5p were cloned into the pmirGLO reporter vector, and a mutated plasmid was used as a control. Cells were cultured in 96-well plates and cotransfected with a miR-335-5p mimic and negative control. After 24 h, the luciferase activity was measured using Envision HTS (PE, USA) according to the manufacturer's protocols.

### 2.10. Statistical Analysis

The statistical analysis was performed with SPSS 22.0 software (Chicago, IL, USA), and the data are presented as the mean ± SEM. Statistical significance was determined using Student's *t*-test for comparisons between the two groups and one-way ANOVA for comparisons of more than two groups. Wilcoxon signed rank tests were applied to analyse the expression of CCRK in tissue samples. A *P* value < 0.05 was considered to indicate a significant difference.

## 3. Results

### 3.1. Upregulation of CCRK Expression in ccRCC Cell Lines and Tissues

**Figure 1:**
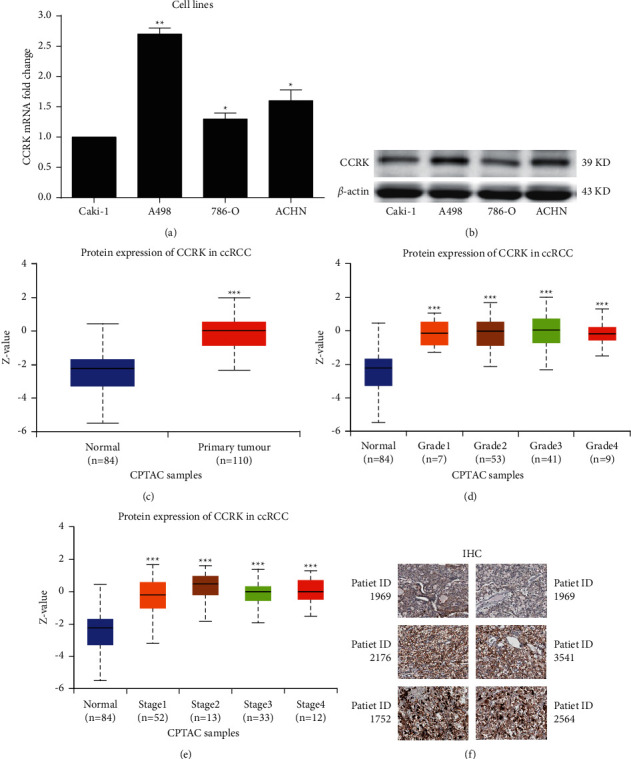
The expression of CCRK was upregulated in the RCC cell lines and tissues. (a) Analysis of the CCRK expression in renal cancer cell lines by RT-qPCR. (b) Analysis of the CCRK protein expression in renal cancer cell lines by western blotting. (c), (d), and (e) Analysis of the CCRK protein expression compared with the normal group based on CPTAC database. (f) Analysis of the CCRK expression in clinical human cancer tissues by IHC. *∗P* < 0.05; *∗∗P* < 0.01; and *∗∗∗P* < 0.001.

CCRK expression in ccRCC cell lines (A498, 786-O, Caki-1, and ACHN cells) was assessed by RT-qPCR. The expression levels of CCRK were significantly upregulated in the ccRCC cell lines (Figure 1(a)), which was further confirmed at the protein level by western blotting (Figure 1(b)). According to the data from the CPTAC database, including 110 ccRCC patients and 84 normal subjects, the relative expression levels of CCRK were analysed by the Wilcoxon signed rank test. Compared with the normal group, CCRK expression was significantly increased in the ccRCC group (Figure 1(c)), and CCRK expression was significantly ascended in different stages (Figure 1(d)), similar results in different grades (Figure 1(e)). Moreover, CCRK expression in human ccRCC tissues were different based on the IHC results, and the majority of ccRCC patients expressed high level of CCRK (Figure 1(f)). These results indicated that CCRK might play an essential role in the pathogenesis of ccRCC.

### 3.2. Proliferation Was Enhanced by CCRK in ccRCC Cells *In Vitro*

**Figure 2:**
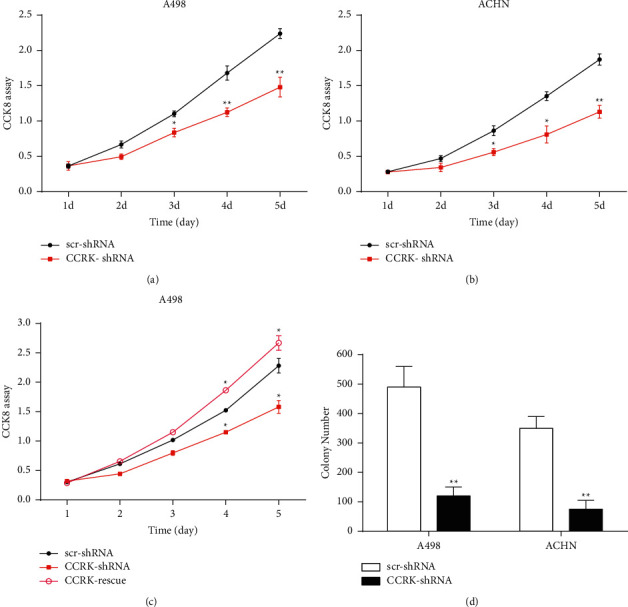
CCRK promoted ccRCC cell proliferation *in vitro*. (a) and (b) Analysis of the CCRK expression and cell proliferation rates in A498 and ACHN cells transfected with scr-shRNA or CCRK-shRNA by western blotting and CCK-8 assay. (c) The CCRK plasmid (pcDNA3.1) was transfected by Lipofectamine 2000 in A498 cells with knockdown of CCRK, CCRK expression and cell proliferation were partly recovered. (d) Colony formation assay in A498 and ACHN cells.

**Figure 3:**
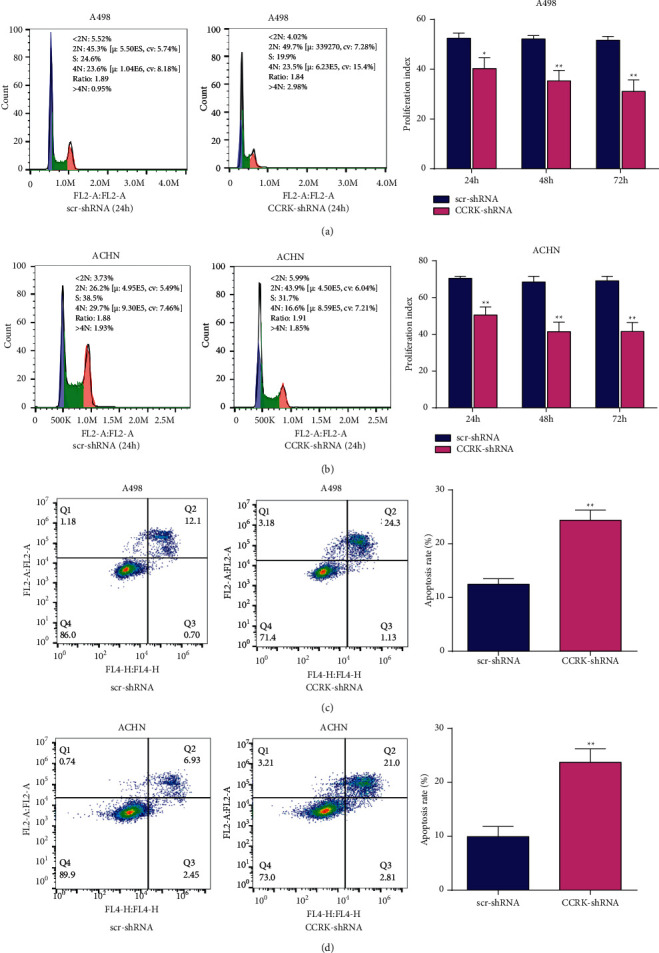
CCRK inhibited apoptosis of RCC cells *in vitro.* (a) and (b) Analysis of the cell proliferation index at 24 h, 48 h, and 72 h in A498 and ACHN cells transfected with CCRK-shRNA or src-shRNA by flow cytometry. (c) and (d) Analysis of the cell apoptosis rates in A498 and ACHN cells transfected with CCRK-shRNA or src-shRNA by flow cytometry.

**Figure 4:**
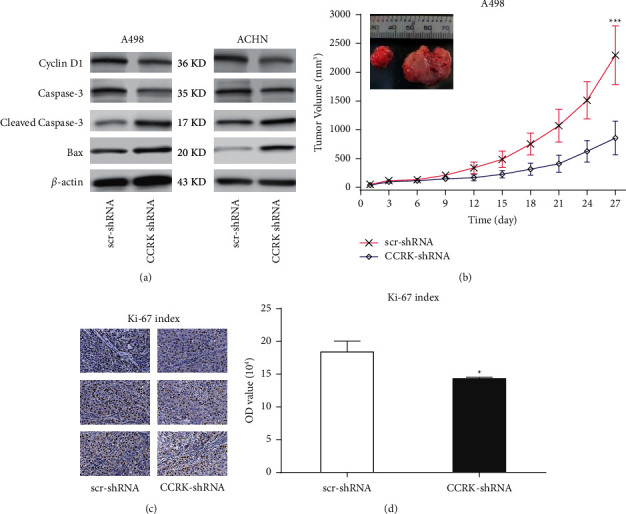
Western blotting *in vitro* and tumour volume analysis *in vivo*. (a) Analysis of the expression of cell cycle marker (cyclin D1) and apoptosis markers (caspase-3, cleaved caspase-3, and Bax) in A498 and ACHN cells transfected with or without lentivirus CCRK-shRNA by western blotting. (b) Analysis of tumour volume *in vivo* after subcutaneous inoculation of nude mice in A498 cells transfected with or without lentivirus CCRK-shRNA (*n* = 4 mice). (c) The IHC stain in tumour tissues of nude mice (*n* = 3). (d) Analysis of optical density (OD) value of Ki-67 index in tumour tissues of nude mice (*n* = 3).

To further explore the potential effects of CCRK on ccRCC cell proliferation, scr-shRNA and CCRK-shRNA were transfected into A498 cells and ACHN cells, respectively. The protein expression levels of CCRK were significantly downregulated in the A498 cells and ACHN cells transfected with CCRK-shRNA compared with the cells transfected with scr-shRNA. The cell proliferation rates of CCRK-shRNA-transfected A498 cells and ACHN cells were significantly decreased compared with those of the scr-shRNA-transfected cells according to the CCK-8 assay (Figures 2(a) and 2(b)). As shown in Figure 2(c), a rescue experiment was performed in A498 cells after CCRK knockdown and CCRK expression was downregulated. However, after transfecting with CCRK plasmid, the CCRK expression and cell viability was partly recovered (Figure 2(c)). Colony formation assays also showed that CCRK-shRNA significantly reduced the colony formation of A498 cells and ACHN cells compared with scr-shRNA (Figure 2(d)). The proliferation index of A498 and ACHN cells treated with CCRK-shRNA was also decreased compared with that of the control cells, as determined by flow cytometry (Figures 3(a) and 3(b)). In addition, the expression of cell cycle and apoptosis markers (cyclin D1 and caspase 3) was drastically reduced in the CCRK-shRNA group compared with the scr-shRNA group *in vitro* (Figure 4(a)). In short, the above results indicated that CCRK plays a tumour-promoting role in ccRCC *in vitro*.

### 3.3. The Activation of Apoptosis was Inhibited by CCRK in ccRCC Cells *In Vitro*

The apoptosis rates of A498 and ACHN cells were detected by flow cytometry to investigate whether CCRK-shRNA could affect the apoptosis of ccRCC cells. The results showed that the apoptosis rate of the cells transfected with CCRK-shRNA was significantly increased compared with that of the cells transfected with scr-shRNA (Figures 3(c) and 3(d)). Then, the expression of apoptosis markers (Bax and cleaved caspase-3) in A498 cells and ACHN cells was significantly increased in the CCRK-shRNA groups compared with the scr-shRNA groups (Figure 4(a)). Collectively, these results revealed that CCRK inhibits apoptosis.

### 3.4. The Tumorigenicity of A498 Cells was Promoted by CCRK *In Vivo*

A498 cells transfected with CCRK-shRNA and scr-shRNA were subcutaneously inoculated into nude mice to assess the function of CCRK *in vivo*. Tumour volume formed in the CCRK-shRNA group was larger than that formed in scr-shRNA group (Figure 4(b)). In Figure 4(c), the percentage of Ki-67 positive cells in the CCRK-shRNA group were significantly lower than that in scr-shRNA group. The optical density (OD) of Ki-67 proliferation index in the CCRK-KD group was significantly lower than that in scr-shRNA group (Figure 4(d), *P* < 0.05). These results revealed that CCRK plays a positive and vital role in the tumorigenicity of ccRCC.

### 3.5. The Bioinformatics Analysis of the Potential miRNAs That Regulate CCRK Expression in ccRCC

**Figure 5:**
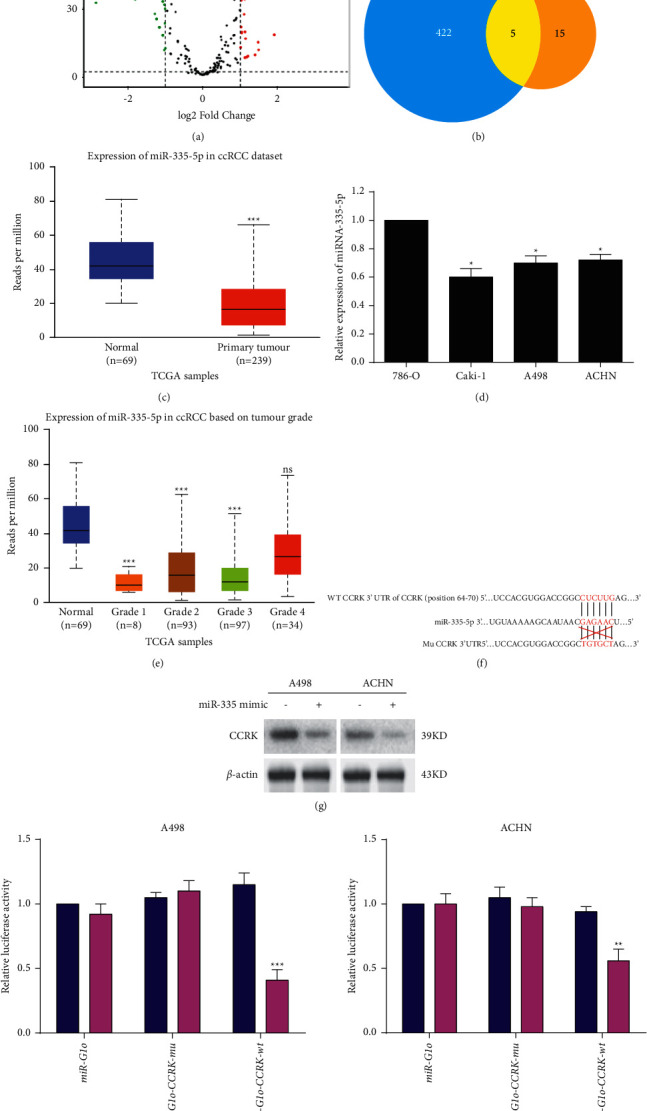
miR-335-5p binding to 3'UTR of CCRK and its effects on ccRCC patients. (a) The volcano plot shows the upregulated (red dot) and downregulated (light-green dot) miRNAs in ccRCC patients and normal patients. (b) The Venn diagram showed the overlapping miRNAs (yellow, 5) between predicted CCRK-targeting miRNAs (blue, 422) by TargetScan 3.1 and downregulated miRNAs (orange, 15) in ccRCC tissues. (c) Comparison of the miR-335-5p expression levels between normal tissues and primary tumour tissues (ccRCC) based on TCGA database. (d) The relative expression of miR-335-5p in RCC cells (A498, 786-O, Caki-1, and ACHN). (e) Comparison of the relative expression of miR-335-5p among tumours of different pathological grades; (f) The miR-335-5p and CCRK 3′UTR had conservative binding sites. (g) The expression of CCRK protein whether adding miR-335-5p mimic into A498 and ACHN cells. (h) Analysis of the luciferase activity between miR-335-5p group and miR-NC group in A498 and ACHN cells transfected with wild-type (wt) or mutant (mu) CCRK 3′UTR. *∗P* < 0.05; *∗∗P* < 0.01; and *∗∗∗P* < 0.001.

Considering that CCRK can affect ccRCC cell proliferation and apoptosis, it has become very important identifying a potential drug or small chemical molecule that can regulate its expression in cancer cells. Increasing studies have reported that miRNAs can play key roles by modulating gene expression to influence cancer progression, which attracted researchers' attention. Here, miRNA sequence data were downloaded from the cancer genome atlas database (TCGA, https://www.cancer.gov/about-nci/organization/ccg/research/structural-genomics/tcga), differentially expressed miRNAs (DEmiRNAs) were analysed with the relevant thresholds *P* < 0.05 and |log2FC| > 1. A total of 149 DEmiRNAs, shown in the volcano plot (Figure 5(a)), were identified between the ccRCC group and the normal group. In addition, the miRNAs that potentially target CCRK were identified using TargetScan (version 3.1). The results displayed that 5 overlapping miRNAs, including miR-335-5p (Table S1), were identified between 422 upregulated miRNAs and 20 downregulated miRNAs in ccRCC (Figure 5(b)). The relative expression of miR-335-5p was significantly downregulated in the ccRCC group compared with the normal group (Figure 5(c)), and miR-335-5p expression was negatively correlated with the CCRK expression, which indicated that miR-335-5p could be a negative predictive factor in ccRCC. Subsequently, the relative expression of miR-335-5p was investigated in A498, 786-O, Caki-1, and ACHN cells, and compared with 786-O cells, miR-335-5p expression was downregulated in A498, Caki-1, and ACHN cells (Figure 5(d)). The further results exhibited that tumour stage (grade I, II, and III) was associated with the miR-335-5p expression in ccRCC patients (Figure 5(e)). In short, these results revealed that miR-335-5p was downregulated in ccRCC and associated with the tumour stage.

### 3.6. The Expression of CCRK Was Regulated by the Binding of miR-335-5p to Its 3′-UTR

To investigate the relationship between miR-335-5p and CCRK expression, we identified a highly conserved site in the CCRK 3′UTR that is targeted by miR-335-5p using the TargetScan database (Figure 5(f)). Subsequently, the western blotting results confirmed that the protein expression levels of CCRK were both downregulated in both A498 and ACHN cells treated with miR-335-5p mimics (Figure 5(g)). To further explore whether miR-335-5p directly binds to the 3′-UTR of CCRK, dual-luciferase reporter assays were performed, which involved wild-type and mutant CCRK 3′UTRs. The relative luciferase activity in A498 and ACHN cells transfected with the wild-type CCRK 3′-UTR was significantly reduced by miR-335-5p transfection, but there was no significant difference between the cells transfected with the CCRK 3′-UTR with mutated miR-335-5p binding sites compared with the cells transfected with miR-NC (Figure 5(h)). Overall, these results indicated that miR-335-5p directly targets and negatively regulates the expression of CCRK.

### 3.7. The Effect of miR-335-5p on ccRCC Cell Proliferation and Apoptosis Can Be Rescued by the Overexpression of CCRK

**Figure 6:**
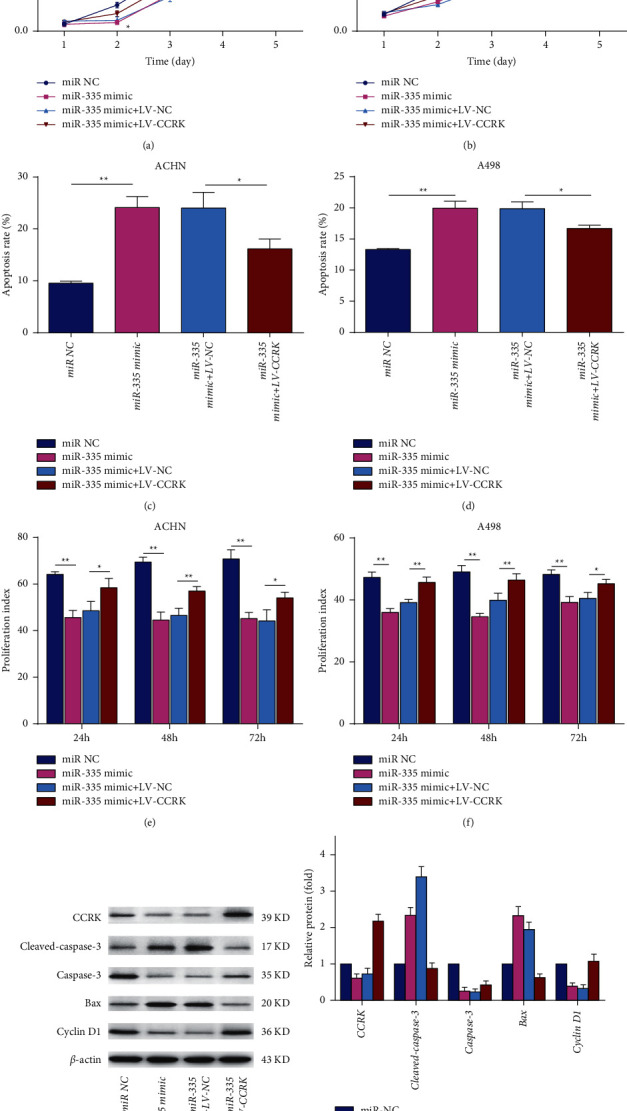
expression of CCRK attenuated the effects of miR-335-5p in ccRCC cell lines. A498 cells and ACHN cells were transfected with miR-335-5p mimic or miR-NC and cotransfected with miR-335-5p mimic + LV-CCRK or miR-335-5p mimic + LV-NC. (a) and (b) Analysis of the cell proliferation rate of 4 groups in A498 cells and ACHN cells by the CCK-8 assay. (c), (d), (e), and (f) Analysis of the apoptosis rates and proliferation index of 4 groups in A498 cells and ACHN cells by flow cytometry. (g), (h), and (i) Analysis of the expression of CCRK, apoptosis markers (caspase-3, cleaved caspase-3, and Bax), and cell cycle marker (cyclin D1) of 4 groups by western blotting. The data are presented as the mean ± SEM. NC, negative control; LV, lentivirus. *∗*/#*P* < 0.05; *∗∗*/##*P* < 0.01; and *∗∗∗*/###*P* < 0.001. *∗*compared with miR NC, ^#^ compared with miR-335 mimic + LV-NC.

Given that miR-335-5p directly targets and negatively regulates the expression of CCRK in ccRCC, CCRK should reverse the effects of miR-335-5p. To test this hypothesis, miR-335-5p mimics or miR-335-5p mimics + LV-CCRK vectors were transfected into A498 and ACHN cells. The results displayed that the cell proliferation indexes were significantly decreased in the miR-335-5p mimic group compared with the miR-NC group, importantly, CCRK can reverse this inhibitory effect of miR-335-5p on ccRCC cell proliferation (Figures 6(a), 6(b), 6(e), and 6(f)). The apoptosis rate of ccRCC cells transfected with miR-335-5p and lentivirus carrying CCRK was similar to the cell proliferation index (Figures 6(c) and 6(d)). In addition, the results confirmed that CCRK expression was decreased in the miR-335-5p mimic group compared with the miR-NC group by western blotting; in contrast, the CCRK expression was notably recovered in the miR-335-5p mimic + LV-CCRK group after ccRCC cells retransfected with CCRK vector (Figures 6(g) and 6(i)). The expression of apoptosis markers (caspase-3, cleaved caspase-3, and Bax) and the cell cycle marker cyclin D1 in ccRCC cells was investigated by western blotting, the results also displayed that CCRK can recover the inhibitory effect of miR-335-5p on ccRCC cell proliferation (Figures 6(g) and 6(i)). Overall, these results confirmed that there are close relationships between miR-335-5p and CCRK in ccRCC.

## 4. Discussion

CCRK is a known nuclear-cytoplasmic shuttling protein containing 11 conserved serine/threonine protein kinase subdomains [6]. CCRK is ubiquitously expressed in the brain and kidney and performs both cell cycle-dependent and cell cycle-independent functions in a wide range of human tissues [6]. In different types of human cancer, including colorectal cancer, hepatocellular carcinoma, lung cancer, medulloblastoma, and ovarian carcinoma, CCRK expression is aberrantly upregulated and plays an oncogenic role [14]. Aberrant expression of CCRK is closely associated with tumour staging, short survival, and poor prognosis [14]. Mechanistically, CCRK is involved in a wide array of cell signalling pathways associated with cell proliferation, which is essential for the genesis and evolution of cancer. For example, downregulation of CCRK-inhibited cell proliferation, caused G1 phase cell cycle arrest, decreased pCdk2 levels, and inhibited Cdk2 kinase activity in HeLa cervical adenocarcinoma cells and human glioblastoma [6, 11, 34]. In addition, CCRK is also involved in the AR and Wnt/*β*-catenin/TCF signalling pathway cascades in human liver malignant neoplasms [35, 36].

In this study, we confirmed that CCRK expression was significantly increased in the ccRCC tissues and several ccRCC cell lines. CCRK promoted cell proliferation and colony formation efficiency and decreased apoptosis *in vitro.* CCRK performs a function in renal cancer that is similar to its function in other cancers. At the protein level, cyclin D1 expression was downregulated when CCRK expression was knocked down. These results suggest that CCRK affects the cell cycle. The decreased proliferation index of CCRK-knockdown ccRCC cells, as observed by flow cytometry, verified this finding. In addition, analysis of apoptosis markers, including caspase-3, cleaved caspase-3, and Bax, also proved the effect of CCRK on cell growth. With xenograft mice models *in vivo*, we also found that CCRK knockdown decreased tumorigenicity. These results show that CCRK could be an oncogene in ccRCC and can be a potential target for cancer therapy.

Furthermore, the expression of CCRK was inhibited by miR-335-5p in ccRCC, and the overexpression of CCRK could partly reverse the antitumour effect of miR-335-5p, which revealed a mechanism of negative regulation between CCRK and miR-335-5p. As prior studies and publications have shown, many miRNAs act as tumour inhibitors or oncogenes in human cancers by regulating the target gene expression [16], and targeting miRNA with mimics or inhibitors could be a possible treatment approach for the clinical therapy. For instance, lncRNA RP11-436H11.5 can regulate the cell proliferation and invasion of RCC by sponging miR-335-5p [30]. Here, our study shows that the expression of miR-335-5p is negatively correlated with the stage of ccRCC in patients. Our results are consistent with prior findings [30], namely, that miR-335-5p expression is downregulated in renal cancer and that the downregulation of miR-335-5p is associated with the disease state (lymph node metastasis, tumour size, and poor T stage) of patients. In brief, our study showed that miR-335-5p was expressed at notably low levels in ccRCC cells and closely associated with the tumour stage, which indicated that miR-335-5p could serve as a promising tumour inhibitor in ccRCC.

## 5. Conclusion

In summary, this work revealed that CCRK was significantly upregulated in ccRCC patients and that knockdown of CCRK-inhibited cancer cell proliferation and enhanced cell apoptosis *in vitro*, which indicated that CCRK could be an oncogene in ccRCC and may be a potential target for cancer therapy in patients with ccRCC. Furthermore, miR-335-5p was negatively related to the CCRK expression. miR-335-5p is downregulated in ccRCC patients and is closely associated with the cancer stage, which reveals that miR-335-5p could serve as a promising tumour inhibitor for the ccRCC therapy.

## Data Availability

Clinical data were downloaded from the CTPAC (https://proteomics.cancer.gov/programs/cptac). miRNA data were analysed online with UALCAN (https://ualcan.path.uab.edu/index.html). The results of immunohistochemistry were obtained from the protein atlas (https://www.proteinatlas.org/ENSG00000156345-CDK20/pathology/renal+cancer). miRNA sequence data were downloaded from TCGA (https://www.cancer.gov/about-nci/organization/ccg/research/structural-genomics/tcga).

## Supplementary Materials

Supplementary Materials Table S1: five overlapping miRNAs between 422 CCRK-targeting miRNAs and 20 downregulated miRNAs in ccRCC tumour tissues.
