# Supplementary material for: Effects of the Targeted Regulation of CCRK by miR-335-5p on the Proliferation and Tumorigenicity of Human Renal Carcinoma Cells
Source: J Oncol. 2022 Oct 14;2022:2960050. doi: 10.1155/2022/2960050 (PMC9586783; doi:10.1155/2022/2960050)
Supplement: Supplementary Materials — Table S1: five overlapping miRNAs between 422 CCRK-targeting miRNAs and 20 downregulated miRNAs in ccRCC tumour tissues. [file 2960050.f1.pdf]

Table **S1:** Five overlapping miRNAs between 422 CCRK-targeting miRNAs and 20 downregulated miRNAs in ccRCC tumour tissues.

**gene_id**	**log2 Fold Change**	**log2CPM**	**P-Value**	**FDR**
hsa-miR-362-5p	-2.560889139	4.60058308	9.51E-109	1.68E-106
hsa-miR-532-5p	-1.254615567	9.895355304	2.36E-43	2.20E-42
hsa-miR-199b-3p	-1.163061108	9.874929973	6.22E-22	2.12E-21
hsa-miR-335-5p	-1.036189295	4.898625145	7.42E-12	1.62E-11
hsa-miR-501-5p	-1.372576966	6.222999456	2.48E-51	3.66E-50
